# Key Considerations for the Use of Seaweed to Reduce Enteric Methane Emissions From Cattle

**DOI:** 10.3389/fvets.2020.597430

**Published:** 2020-12-23

**Authors:** Sandra Vijn, Devan Paulus Compart, Nikki Dutta, Athanasios Foukis, Matthias Hess, Alexander N. Hristov, Kenneth F. Kalscheur, Ermias Kebreab, Sergey V. Nuzhdin, Nichole N. Price, Yan Sun, Juan M. Tricarico, Adele Turzillo, Martin R. Weisbjerg, Charles Yarish, Timothy D. Kurt

**Affiliations:** ^1^World Wildlife Fund, Washington, DC, United States; ^2^Land O'Lakes Inc., Arden Hills, MN, United States; ^3^Foundation for Food and Agriculture Research, Washington, DC, United States; ^4^Bigelow Laboratory for Ocean Sciences, East Boothbay, ME, United States; ^5^Department of Animal Science, University of California, Davis, Davis, CA, United States; ^6^Department of Animal Science, The Pennsylvania State University, University Park, PA, United States; ^7^US Dairy Forage Research Center, USDA-Agricultural Research Service, Madison, WI, United States; ^8^Section of Molecular and Computational Biology, University of Southern California, Los Angeles, CA, United States; ^9^Cargill Animal Nutrition and Health, Elk River, MN, United States; ^10^Innovation Center for US Dairy, Rosemont, IL, United States; ^11^Department of Animal Science, Aarhus University, AU-Foulum, Tjele, Denmark; ^12^Department of Ecology & Evolutionary Biology, The University of Connecticut, Stamford, CT, United States

**Keywords:** methane, cattle, seaweed, dairy, beef, agriculture, livestock, ruminant

## Abstract

Enteric methane emissions are the single largest source of direct greenhouse gas emissions (GHG) in beef and dairy value chains and a substantial contributor to anthropogenic methane emissions globally. In late 2019, the World Wildlife Fund (WWF), the Advanced Research Projects Agency-Energy (ARPA-E) and the Foundation for Food and Agriculture Research (FFAR) convened approximately 50 stakeholders representing research and production of seaweeds, animal feeds, dairy cattle, and beef and dairy foods to discuss challenges and opportunities associated with the use of seaweed-based ingredients to reduce enteric methane emissions. This *Perspective* article describes the considerations identified by the workshop participants and suggests next steps for the further development and evaluation of seaweed-based feed ingredients as enteric methane mitigants. Although numerous compounds derived from sources other than seaweed have been identified as having enteric methane mitigation potential, these mitigants are outside the scope of this article.

## Introduction

Enteric fermentation is the highly evolved process that allows ruminants to digest cellulose, the basic component of plant cell walls. Rumen microbes ferment simple and complex carbohydrates like cellulose to produce volatile fatty acids (VFAs), which can satisfy over 70% of the energy requirements of the host animal (1). However, the production of certain VFAs also produces hydrogen (H_2_), which is converted to methane (CH_4_) by methanogenic archaea (i.e., methanogens). Although CH_4_ is short-lived relative to other GHG, persisting in the atmosphere for about 10 years, it has a significant impact on the climate due to its global warming potential (GWP), which is ~28-times higher than that of carbon dioxide (CO_2_) (2, 3). Enteric methane, not to be confused with methane emissions from manure, is mostly released by eructation directly from the animal and is the largest direct contributor to GHG emissions in beef and dairy production (4). In the U.S., an estimated 26.7% of total CH_4_ emissions are attributed to enteric fermentation, which corresponds to approximately 2.7% of anthropogenic GHG emissions (5).

Numerous approaches for mitigating enteric methane emissions have been proposed and investigated over the last several decades, and these primarily focus on animal nutrition, genetics, and management (6). On the global scale, improving animal efficiency is perhaps the most effective methane mitigation strategy. Feed additives, such as the inhibitor 3-nitrooxypropanol (3-NOP), have been shown to consistently decrease enteric methane emissions by up to 30% in both dairy and beef cattle (7, 8). However, their use in ruminant diets is currently not widespread due to the need for regulatory approval, the lack of incentives such as carbon credits or gains in animal productivity, and the absence of legislative mandates for agricultural GHG reduction in most regions.

Feeding livestock many seaweeds—also known as red, green or brown marine macroalgae—has been shown to reduce methane production, but with highly variable results (9–12). For example, *in vitro* analysis suggested that the tropical/subtropical red seaweed *Asparagopsis taxiformis* can reduce methane production by 95% when added to feed at a 5% organic matter inclusion rate (13). An *in vivo* study in dairy cows using *A. armata*, a closely related species, showed that methane production and yield (adjusted for feed consumption) decreased 67 and 43%, respectively, at a 1% level of dry matter inclusion in the diet (13). Kinley et al. (14) reported that inclusion of *A. taxiformis* at 0.10 and 0.20% of dietary dry matter over a 90 day period decreased methane production in steers up to 40 and 98%, and produced weight gain improvements of 24 and 17 kg, respectively, relative to control steers. These findings are both remarkable and need to be replicated by other research teams. Brown seaweeds like *Ascophyllum nodosum* have a measurable, but less substantial, impact on enteric methane at higher dietary inclusion rates (15). The efficacy of methane reduction appears to correlate with the concentration of bromoform compounds, which appear to be the main active ingredients although other yet to be identified substances may contribute to methane reduction as well. The concentration of bromoform in *Asparagopsis* was 6.55 mg/g in the study conducted by Kinley et al. (14) compared to 1.32 mg/g in the study by Roque et al. (13).

Livestock have grazed on beach cast seaweeds for millenia and seaweeds have been foraged for use as feeds in coastal communities around the globe (16, 17) with evidence of intentional feeding in Greece dating to at least 100 BC (18, 19). Today, most algae-based livestock feed additives are made from milled or ensiled brown seaweeds such as kelp (cf. *Laminariales*) and rockweed (cf. *A. nodosum)* (19). Seaweeds provide an array of essential nutrients as well as numerous secondary plant compounds. Certain seaweeds also contain omega-3, omega-6 and other polyunsaturated fatty acids (PUFAs) (16, 20, 21) which could aid ruminant reproduction through improved conception rates and reduced pregnancy losses (22). While the mechanisms by which PUFAs aid reproductive success are unclear, fatty acid compositional analyses suggest it may be related to the incorporation of PUFAs into reproductive organ tissues (22). Feeding ruminants brown seaweed extracts may have health benefits related to reducing oxidative stress, stress markers and incidence of ketosis (23–25). Feeding kelp may also improve cattle performance, as trials in lactating dairy cows have demonstrated significant reductions in ruminal ammonia-nitrogen and increased acetate, propionate, and total volatile fatty acids (26) - effects that may improve milk yield (16). In addition, seaweeds that mitigate enteric methane, such as *Asparagopsis* sp., appear to shift hydrogen metabolism to propionate production, enabling greater energy utilization from feedstuffs consumed by ruminants (14). Algae-based feeds may also improve fatty acid profile (26–32), increase fat content and reduce somatic cell counts in milk (33). However, seaweeds may also contain inorganic elements and heavy metals like iodine, bromine, arsenic and other halogenated bioactive organic compounds that at high levels may cause toxicity in animals and humans (19, 34). Chronic excess iodine intake from consumption of kelp meal in dairy cows can lead to iodine-enriched milk (21, 23, 35, 36). Interestingly, this could present an opportunity to address insufficient iodine intake in humans that is estimated to impact 2 billion individuals globally (37). However, it is also important to consider the need to avoid excess iodine consumption in humans, especially in populations where iodine intake is already at sufficient levels. There are also safety concerns regarding trihalomethanes such as bromoform, the main active ingredient in the methane-inhibiting *Asparagopsis* species. The U.S. EPA has established a 0.08 mg/L limit for trihalomethanes in drinking water (38), so any levels above this limit in milk from cows fed seaweed could present a human food safety concern.

Today, widespread use of seaweed as a livestock feed ingredient would require large-scale, intensive farming to produce the volumes needed for the feed industry. This could present several challenges. Environmental concerns regarding ocean seaweed farming include the potential for marine mammal entanglement, larval transport and escaped lines or rigs subsequent to storm damage (39). It will be important to identify and select seaweeds that are native to the farming region, or that pose little-to-no risk of environmental impact. In the near future, it may be possible to grow seaweeds under controlled (i.e., nearshore or indoor) conditions, with production of bioactive compounds through bioengineering approaches.

However, ocean seaweed farming can also benefit the environment in many ways. In oceans, seaweed provides rich habitat for fishes, sequesters carbon, removes excess nutrients and protects calcifiers from acidification (40–47). On shorelines, seaweed provides protection from wave action and supplies feed for many native species (48). Seaweeds can also be cultivated in recirculating, land-based aquaculture systems and are often used for biofiltration in multi-trophic recirculating systems to maintain water quality (49–53).

### Seaweed Research and Production

To supply the volumes of seaweed that would be needed by the beef and dairy industries, strategies that enable the efficient and sustainable production of seaweeds of consistent quality need to be developed through aquaculture. The first challenge will be to identify the ideal cultivars, or blends thereof, that possess both the qualities desired by end-users (i.e., potent methane reduction, safety, palatability, etc.) as well as the ability to be cultivated at scale. In addition, there is a need to understand how location, season, breeding, processing and other factors impact quality and consistency of the end product. In many countries, a consistent, streamlined, inter-state policy framework for seaweed production is needed to provide regulatory certainty and encourage investment and industry expansion (54, 55). Policy researchers, ecologists, and regulatory agencies, in collaboration with seaweed farmers, must also consider social license to operate, particularly in nearshore environments where coastal landowners may perceive seaweed farming as an activity that would negatively impact property values (56).

Regulatory issues also inform the feeding of seaweeds to livestock. Currently, the U.S. Food and Drug Administration (FDA) limits the use of seaweed in livestock diets to select seaweed species, uses and inclusion rates (See AAFCO 57.73, 60.19, 60.76 and Title 21 Code of Federal Regulations 582.30 and 582.40).^1^ In order to expand the use of seaweed in livestock diets in the U.S., more extensive research is needed on currently approved, as well as new, seaweed sources to demonstrate their safety and efficacy in accordance with FDA regulations.

Due to its high water content, seaweeds are typically solar dried prior to shipping and long-term storage. Processing may impact the bioactive compounds in seaweed; for use in livestock feed, the active ingredients will need to be stable in high heat, liquid, long-term storage and in combination with vitamins, minerals, and other compounds (57). Energy requirements and life-cycle assessments (LCA) need to be conducted for the different aspects of seaweed production including cultivation and harvesting, drying, processing, and distribution.

One of the most pressing research needs is to develop appropriate methods for screening the diversity of species that may mitigate enteric methane emissions, prior to investing significant time and money in cultivation and/or animal trials. Thus, researchers with an interest in studying seaweeds for enteric methane mitigation are encouraged to develop agreed-upon guidelines for evaluating seaweeds *in vitro* (see Table 1). These methods should enable *in vivo* dosing recommendations as they relate to active compounds in seaweed, which can vary significantly between species (21, 57). However, it is currently difficult for scientists to obtain the sufficient biomass from many seaweed species to conduct *in vivo* trials with sufficient animal numbers, replicates and study duration. Collaborative teams of aquaculturists, seaweed biologists and crop breeders will need to envision scalable and economic approaches to seaweed cultivation and breeding, coupled with *in vitro* and *in vivo* evaluation, to inform farming practices and species selection for research and development (58).

**Table 1:** Key considerations and research recommendations for the development and evaluation of seaweed as an enteric methane mitigant.

	**Key considerations**	**Research recommendations**
Seaweed production	• Environmental ° Water quality and habitat improvement ° Carbon sequestration ° Entanglement issues ° Invasive species introduction ° Carbon footprint ° Spread of diseases • Potential markets/return on investment ° Use of co-products in other industries ° Competitiveness in marketplace • Biological ° Seed banks and breeding programs ° Phenotyping/trait selection ° Processing, storage and transportation techniques that maintain biological activity • Geophysical • Water conditions amenable to cultivation • Heavy metal and other contaminant levels • Regulatory • Site permitting and social license to develop farms • Training and capacity building	• Functional annotation of seaweed genomes • Agreed-upon protocols for *in vitro* analysis (including preparation of fermentation assays) • Optimal methods for seaweed processing (e.g. drying, freezing, fermentation, extraction of bioactives, etc.) • Impact of different cultivation and processing techniques on iodine and heavy metal concentrations • Investigation of co-products, alternative uses of biomass before/after bioactive extraction • Siting and environmental impact assessments
Animal Feed Production	• Environmental ° Waste materials/gases generated during processing and distribution ° Carbon footprint • For processing • For distribution • Potential markets/return on investment ° Use of co-products in other industries ° Competitiveness in marketplace ° Cost of seaweed products and price at farmgate • Quality, consistency and reliability of ingredients ° Active compound levels ° Processing, storage and transport techniques that maintain biological activity ° Nutrient profile ° Heavy metals and other contaminant levels • Regulatory • Process, expense, and duration of getting FDA/AAFCO approval for applying seaweed products in animal feed • Guidance on approval and labeling of feed ingredients vs. additives	• Standard protocols for consistent, comparative evaluation of active compound levels in seaweed products • Standard protocols for potential contaminants and nutritional profiles of seaweed products • Effects of feed processing on content of active compounds and nutritional profile, optimized for large scale manufacturing • Develop Safety Data Sheets for handling, processing and transporting seaweed products • Develop protocols for monitoring potential environmental issues during processing and transportation of seaweed product • Market analysis of seaweed products
Livestock production	• Environmental ° Reliable long-term efficacy in different stages of production, geographies and farming systems ° Impacts on manure products (e.g., biogas, nutrients) and land application of animal wastes • Potential markets/return on investment ° Cost/benefit of the feed ingredient ° Potential premiums/branded products • Health and productivity ° Long-term animal health and safety ° Meat and milk product quality and safety ° Production volume, feed and growth efficiency ° Interaction with other feed ingredients • Feasibility of application ° Palatability ° Formulation and delivery to animals in different production systems	• Validated methods for measuring enteric methane missions • Mechanism of action, specificity, effect on gut microbiome • Efficacy, dose, duration of feeding, residues and withdrawal time • Duration of studies, e.g. through one lactation or through feeding program • Stage of production, e.g. lactating vs. dry cow; pasture vs. feedyard • Consideration of animal age, weight, sex, breed and genetic background • Animal health and wellbeing: morbidity, mortality, lameness, mastitis, etc. • Animal efficiency (growth, milk production, feed conversion) • Meat and milk product safety, nutrients composition, digestibility and energy value • Compounds excreted in manure and effect on environment and biogas production • Product quality, taste/sensory evaluation
Food service/retail	• Understanding of potential environmental, social and economic risks and benefits across the supply chain • Awareness of investment opportunities (including co-products) to advance scaling of seaweed-based feed ingredients • Insights into consumer acceptance of seaweed-based feed ingredients and onshore/nearshore/offshore aquaculture • Full life-cycle assessment	• Clear, concise, consistent, and globally accepted metrics for full lifecycle analysis and risk assessment • Full lifecycle analysis of processing and transporting seaweed products • Ability to enter carbon and nitrogen markets • Communications, marketing and education approaches • Economics and potential return on investment, including premiums and branded products • Comparative studies of seaweed-based feed ingredients against other enteric methane suppressants or health feed additives available on the market

**Figure 1:**
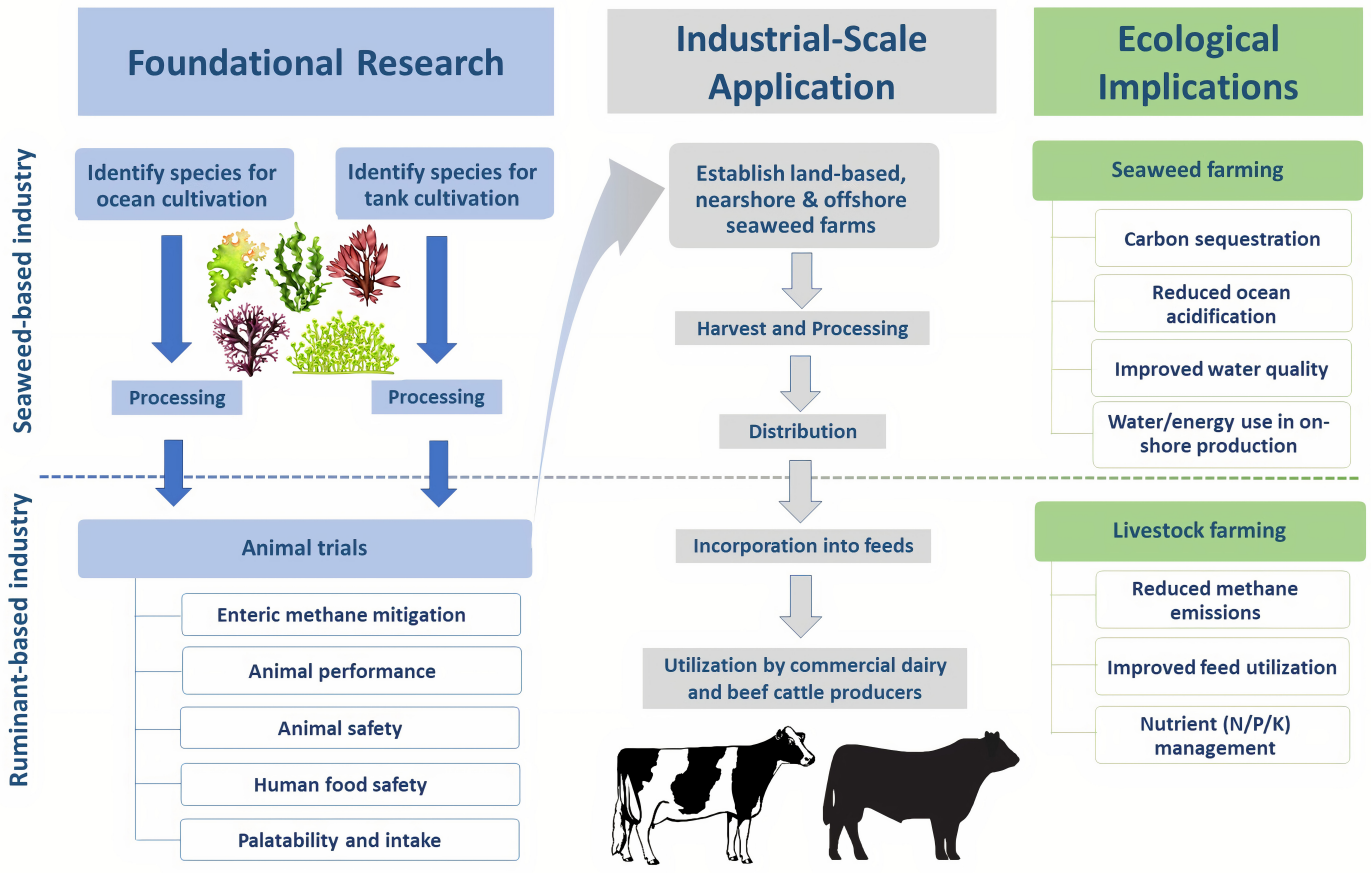
Proposed pathways to development of seaweed products for enteric methane mitigation.

In addition to enteric methane mitigation, there are potential opportunities for seaweed producers to capitalize on ecosystem services or carbon trading markets, as well as co-products that can be used in biofuels, cosmetics, fertilizers and other industries (41–43, 53, 59–63). Alternatively, seaweeds can be produced as part of an integrated aquaculture production system alongside other cultured species such as shellfish and finfish, and some can be grown for human consumption (31, 46, 49, 64, 65). However, several major obstacles need to be overcome for future seaweed farmers to realize any of these additional benefits of producing a methane mitigant for livestock (Figure 1).

### Animal Feed Research and Production

Large-scale animal feeding operations and feed manufacturers require consistent volume, quality and safety of raw materials. In addition, new feed ingredients must not displace critical protein, carbohydrates, minerals, and other nutrients in the diet. Therefore, it is likely that seaweeds would need to be used as feed additives, which are generally defined as <1% of the dry matter intake (DMI) of an animal's diet. Even as a feed additive, supplying the livestock industry with sufficient seaweed may be a challenge. Globally, seaweed farming generates more than 30 million metric tons (MMT, wet weight) of material^2^ (44, 66) which, assuming 80–90% water content, translates to ~3–6 MMT when dried. Based on typical dry matter intake for beef and dairy cattle, we calculated the potential volume of seaweed that would be needed to supply the 93 M U.S cattle at a 1% inclusion level:

93 M cattle × 9–10 Kg DMI per animal × 365 days per year = 305–339 MMT DMI per year
305–339 MMT dry matter per year × 0.01 seaweed inclusion level **≈** 3–3.4 MMT dry seaweed per year

The estimated 3–3.4 MMT of dried seaweed required per year would represent over half of all seaweed currently produced globally. Supplying the global herd −1.4 billion cattle—would not be feasible, however it is presumed that widespread use of methane mitigants in smallholder or subsistence production systems is unlikely to occur due to cost and other limitations.

In addition to quality control issues related to the consistency and activity of seaweed raw materials, producers will need to determine best methods for drying or otherwise reducing the large amounts of water contained in fresh seaweed, as the wet weight would present a major challenge to transporting seaweed from production sites to processing facilities. Feed manufacturers will need to consider any special encapsulation or protective methods that might be needed for delivery and to increase shelf life, in addition to the by-products that will be produced and how to reuse waste materials.

One of the greatest challenges feed manufacturers face in developing seaweed-based feed ingredients is compliance with industry regulations. Animal feed ingredients are highly regulated in many countries, with approval processes similar to foods for human consumption. In the U.S., feeds must typically meet animal and human safety standards, with thorough documentation of supporting data and methods used to obtain it, as well as detailed descriptions of the manufacturing process, toxicology of any potentially harmful substances in the ingredient, proof of consistent manufacturing, and a proposed legal definition for the ingredient (see footnote 1) (34, 67). In addition, feed manufacturers must find ways to produce seaweed-based feed ingredients that are compatible with the feed manufacturing and handling systems used by the industry today. For example, seaweed products for the animal feed industry need to be in dry form for inclusion into mixed feed ingredients, have concentrated active compounds to allow for low inclusion rates, have sustained activity over time to meet reasonable shelf life requirements of the ingredient alone and in mixed feeds, be able to withstand transportation and processing such as pelleting or extrusion, be palatable to animals, have a known and consistent nutrient profile, and be cost effective for the feed manufacturers and their customers. Finally, since quality and effectiveness of a seaweed product may be modified during the feed production, storage and mixing processes, the efficacy, safety, and stability of the final formulation must be evaluated prior to commercial application.

### Livestock Research and Production

Use of seaweeds for enteric methane mitigation in a commercial setting is still largely untested. Several *in vivo* studies have evaluated the effects of a specific seaweed, *Asparagopsis* sp., on methane emissions and productivity of sheep, beef and dairy cows. However, it is unclear whether the observed effects are repeatable in the long-term and across different production systems. Additionally, the seaweed materials used in these studies were harvested from the wild (i.e., not farmed seaweed), and the content of active compounds was not consistent (13, 14, 68). Currently, no *Asparagopsis* sp. supply chain is available for livestock feed and the feasibility and costs for scaling production of these species are yet to be determined.

Variability among *in vivo* research trial designs has made it challenging to build a comprehensive dataset describing the impacts of feeding seaweed to livestock. Future *in vivo* studies need to demonstrate not only that feeding seaweed products has no deleterious health impacts in animals and that foods for human consumption are safe, but also that the products are effective when fed long-term. Research is also needed to compare seaweed-based products with other methane mitigants currently available in the marketplace, including several microbial-based and plant-based oil products that make off-label methane reduction claims which have not been approved by global regulatory bodies. This will be particularly important as more methane inhibitors become commercially available and methane emissions become subject to regulation at the farm level.

Both short- and long-term animal trials are needed to comprehensively evaluate the use of seaweed in cattle production. Short-term studies should determine which seaweed species have the greatest potential to reduce methane. These studies should also evaluate impacts on animal productivity (e.g., beef and milk production), feed intake, animal health, product quality, active compound residue in edible food products and potential changes in manure composition. Further, as both polymeric and simple carbohydrates in seaweed are very different from land-grown feedstuffs, studies on the digestibility and energy value of these carbohydrates for cattle production is required. Longer-term animal trials are also needed to further evaluate the effects of selected seaweeds on methane emissions, productivity, health, product quality, digestibility of nutrients, active compound residues in manure, and manure GHG emissions. Feeding approaches for seaweed products will need to accommodate differing production systems, as effects may be influenced by breed, diet, climate, geography, and other variables. Development of a commercial seaweed feed product must include mitigation of any safety or environmental hazards, including the presence of halogenated compounds.

The bioactive compounds in seaweed responsible for reducing enteric methane emissions appear to act by modulating rumen microbial populations (69). Research in humans has shown that seaweed can act as a prebiotic, positively affecting the composition and function of the gastrointestinal microbial community, commonly referred to as the gut microbiome (70–72). Since our knowledge of the microbiome and its contribution to animal health is still in its infancy, metagenomic studies are imperative to understanding how certain seaweeds impact the rumen microbiome and whether these effects could be manipulated to benefit animal health and productivity, as well as the environment.

For livestock producers, it is important to evaluate the economic benefits of any future seaweed product. Even if regulations mandate the use of seaweed or other products to reduce methane emissions, farmers' financial burden could increase if animal performance is not improved simultaneously (e.g., improved productivity, efficiency, health, or product quality). The value of the improvement must therefore be enough to cover the cost of the product or additional incentive programs will need to be established to achieve widespread adoption.

### Food Service and Retail

Climate change has become a major global threat that many food and beverage companies are compelled to address, either by reducing GHG emissions associated with their operations and electricity consumption (Scope 1) or by reducing indirect emissions that occur within their supply chain (Scope 3; https://ghgprotocol.org). Over 850 companies have set science-based targets to reduce GHG emissions from their operations and supply chains to meet the goal of limiting warming to 1.5°C above pre-industrial levels as set out by the Paris Agreement (https://sciencebasedtargets.org).

Successfully reducing enteric methane emissions from cattle using seaweeds may help alleviate consumer concerns about the climate impacts of animal production and increase consumer acceptance of animal-source foods. In addition to meeting climate goals, seaweed could offer a path to a “carbon-neutral” label that may have added value in the marketplace. Farmers may benefit financially from a premium for these animal products or through carbon trading once ecosystem services markets are well established (43, 59, 60, 73). However, a lack of science-based evidence regarding potential risks and benefits of seaweed-based feed ingredients is limiting corporate investment in research and development. In addition, consumer insights are needed to understand potential acceptance of seaweed-based feed ingredients, as well as willingness to pay for meat and dairy products that are produced with these ingredients. A comprehensive framework to assess the environmental, economic and social sustainability of seaweed-based animal feeds is needed and would broaden the scope of evaluation beyond the traditional lens of animal health, productivity and profitability. There is significant potential for the development of marketing strategies that appeal to consumers' desire to contribute to low carbon economies and lifestyles, in which products—including meat and dairy—are positioned as sustainably sourced foods.

## Discussion

Results of initial *in vitro* research trials indicate that feeding small amounts of seaweed to cattle may reduce the production of enteric methane. Multiple *in vitro* research projects are currently underway to determine the methane reduction potential of a wide variety of seaweeds, setting the stage for further investigation of promising candidates *in vivo*. Stakeholders from the animal, feed, seaweed, food and beverage industries, along with research and funding organizations, have identified many outstanding questions that need to be answered to fully evaluate seaweed-based feed ingredients as a long-term, sustainable solution to enteric methane emissions. We encourage researchers, supply chain managers and specialists in consumer behavior to work collaboratively to develop research methods that deliver consistent, replicable and comparable results, to share knowledge and resources, and to embark on an accelerated journey to find answers to the outstanding questions presented here.

## Data Availability Statement

The original contributions presented in the study are included in the article/supplementary materials, further inquiries can be directed to the corresponding author/s.

## Author Contributions

TK, MH, EK, SN, and NP contributed to the Figure. TK organized the manuscript sections, finalized and formatted the authors' text and references, and submitted the manuscript. All authors contributed to writing and revising the manuscript text and table.

## Conflict of Interest

DC and YS were employed by the companies Land O'Lakes Inc. and Cargill Inc., respectively. The remaining authors declare that the research was conducted in the absence of any commercial or financial relationships that could be construed as a potential conflict of interest. The reviewer MH declared a past co-authorship with the authors AN and EK to the handling editor. The handling editor declared a past co-authorship with one of the authors EK.

## Footnotes

^1^Association of American Feed Control Officials (AAFCO). (2020). https://www.aafco.org/Publications.

^2^Food and Agriculture Organization of the United Nations (FAO). (2020). Fishery Statistical Collections. Global Aquaculture Production. http://www.fao.org/fishery/statistics/global-aquaculture-production/en (Accessed 06/15/2020).
